# Risk Factors for Perioperative Hemodynamic Instability in Pheochromocytoma: A Systematic Review and Meta-Analysis

**DOI:** 10.3390/jcm10194531

**Published:** 2021-09-30

**Authors:** Fumihiko Urabe, Shoji Kimura, Kosuke Iwatani, Kazuhiro Takahashi, Kagenori Ito, Kojiro Tashiro, Shunsuke Tsuzuki, Jun Miki, Takahiro Kimura, Nozomu Furuta, Shin Egawa

**Affiliations:** 1 Department of Urology, The Jikei University School of Medicine, Tokyo 105-8461, Japan; shoji.kimura-1228@hotmail.co.jp (S.K.); ne.jp.ne.jp.ne.jp.ne.jp@gmail.com (K.I.); kazu-negi@hotmail.co.jp (K.T.); kagito@ncc.go.jp (K.I.); tashikoji@gmail.com (K.T.); tsuzushun60@gmail.com (S.T.); junmiki.jikei@gmail.com (J.M.); tkimura0809@gmail.com (T.K.); nozomu21@jikei.ac.jp (N.F.); s-egpro@jikei.ac.jp (S.E.); 2 Department of Urology, The Jikei University Kashiwa Hospital, Chiba 277-8567, Japan

**Keywords:** pheochromocytoma, adrenalectomy, hemodynamic instability, hypertension, hypotension

## Abstract

Objective: To evaluate the risk factors of perioperative hemodynamic instability in pheochromocytoma, we conducted a systematic search of the literature using the Preferred Reporting Items for Systematic Reviews and Meta-analysis. Methods: In April 2021, we systematically searched PubMed, the Cochrane library, and Scopus for relevant studies on the risk factors of perioperative hemodynamic instability of adrenalectomy in patients with pheochromocytoma, and we subjected the findings from those studies to formal meta-analysis. Results: Our systematic review identified 14 studies involving 1725 patients, of which nine studies with 967 patients were eligible for meta-analysis. The results of meta-analysis showed that tumor size (odds ratio (OR): 1.14 for each increased cm, 95% confidence interval (CI) 1.03–1.26, z = 2.57) and urinary norepinephrine (OR, 1.51: 95% CI 1.26–1.81; z = 4.50) were most closely associated with the occurrence of perioperative hemodynamic instability. Conclusion: These findings suggest that tumor size and urinary norepinephrine are important predictors and risk factors for perioperative hemodynamic instability in adrenalectomy for pheochromocytoma. Such findings may be of value to surgeons and anesthesiologists when considering or preparing for this procedure.

## 1. Introduction

Adrenal pheochromocytoma is defined as a tumor derived from catecholamine-producing chromaffin cells in the adrenal medulla. Almost all adrenal pheochromocytomas produce, store, release, and metabolize catecholamines and can cause life-threatening systemic effects such as, stroke, heart attack, and multiple organ failure [1].

Although resection is the only curative treatment for pheochromocytoma, this surgery carries a very high risk of eliciting massive catecholamine release, which can cause severe hypertension [2,3]. Hypotensive episodes can also occur after tumor resection, requiring the sustained administration of vasopressor agents in addition to aggressive volume expansion. These perioperative hemodynamic instabilities can sometimes occur even when adequate medications before surgery have been provided [4].

To date, although several researchers have investigated possible risk factors for perioperative hemodynamic instability in pheochromocytoma, no systematical evaluation has been performed [5,6,7,8]. We therefore conducted a systematic review and meta-analysis of literature to assess current thinking on the risk factors for the occurrence of perioperative hemodynamic instability in patients with pheochromocytoma.

## 2. Methods

### 2.1. Search Strategy

Our systematic review and meta-analysis were based on the requirements of the Preferred Reporting items for Systematic Review and Meta-analysis (PRISMA) statement [9]. The protocol was preregistered in the international Prospective Register of Systematic Reviews database. We first searched the PubMed, Cochrane Library, and Scopus electronic databases on 15 April 2021 for studies published through March 2021, screened all study titles and abstracts, and then assessed the eligibility of the candidate full-text articles. Two investigators (F.U. and S.K.) independently extracted data and checked the appropriateness of each article in full text review. Disagreements were resolved by consensus with a third investigator or by the decision of the senior author (S.E.). Key words in the search were “pheochromocytoma” AND “adrenalectomy” AND “hemodynamic instability” OR “hypertension” OR “hypotension”. Our primary outcome of interest was the occurrence of hemodynamic instability intra- or post- adrenalectomy.

### 2.2. Selection Criteria

Eligible studies were those that compared pheochromocytoma patients who experienced hemodynamic instability to those who did not, either during or after surgery, with the objective of assessing the relationship between risk factors and hemodynamic instability, utilizing univariate and multivariate logistic regression analysis in cohort studies. We excluded articles that were published in a language other than English, reviews, commentaries, and case series. If multiple articles were published by the same group using similar cohorts, we selected either the more recent or the higher quality publication.

### 2.3. Data Extraction

Two investigators (F.U. and S.K.) worked independently to extract the required data. Data included the first author’s name, publication year, country in which patients were enrolled, period of enrollment, number of patients, age, tumor size, and risk factors. Odds ratios (ORs) and 95% confidence intervals (CIs) were determined for risk factors associated with occurrence of hemodynamic instability. All discrepancies related to data extraction were resolved in a consensus meeting.

### 2.4. Quality Assessment

After selecting the studies for inclusion, we assessed the quality of each study on the Newcastle-Ottawa Scale [10], based on the Cochrane Handbook for systematic reviews [11]. The scale uses a 0–9 scale and focuses on three factors: Selection (1–4), Comparability (1–2), and Exposure (1–3). The main confounders were identified as important prognostic factors for hemodynamic instability. The presence of confounders was determined by a consensus and review of the literature. Those studies with scores above 6 were considered “high-quality” choices.

## 3. Statistical Analysis

A forest plot was used to assess ORs from the multivariate logistic regression analyses of individual studies and to obtain a summary OR for the relationship between risk factors and hemodynamic instability. If the study reported only the OR and *p*-value, we calculated the 95% CI [12,13]. The Cochrane Q test and I^2^ statistics were used to evaluate heterogeneity among outcomes of the studies in this meta-analysis, with significant heterogeneity indicated by *p* < 0.05 in the Cochrane Q test and ratio >50% in I^2^ statistics and with the use of random effect models based on the DerSimonian and Laird method [14,15,16]. We used fixed-effect models to calculate pooled ORs for non-heterogeneous results and funnel plots to assess publication bias. All statistical analyses used Stata/MP 14.2 (Stata Corp., College Station, TX, USA). Statistical significance was set at *p* < 0.05.

## 4. Results 

### 4.1. Study Selection and Characteristics

**Figure 1:**
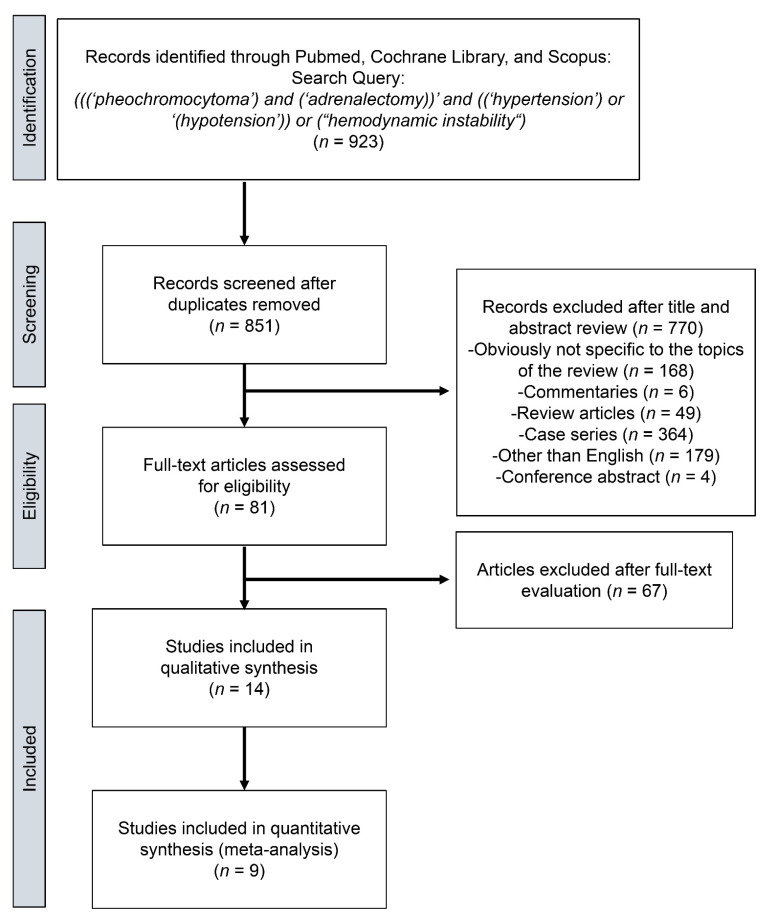
Preferred Reporting Items from the Systematic Review and Meta-Analysis (PRISMA) flow chart showing the process of article selection to analyze risk factors for hemodynamic instability in pheochromocytoma.

We identified a total of 923 articles from the search query. Of those, 72 duplicates were removed, and 770 articles were excluded after initial screening and abstract review. Sixty-seven additional articles were excluded after full-text evaluation. The remaining 14 articles were subjected to systematic review [4,5,6,7,17,18,19,20,21,22,23,24,25,26], and nine of those articles were meta-analyzed [4,6,7,17,19,21,22,23,24]. We detailed the study selection process in a flow chart (Figure 1). The extracted data from the 14 studies are summarized in Table 1, Table 2 and Table 3. All included studies were of retrospective design and were published between 2014 and 2019: three studies from North America, three studies from Europe, seven studies from Asia, and one with international collaboration. The range of age and tumor size were 38.6–54 years and 3.8–6.5 cm, respectively. Of the studied patients, 757 were male and 768 were female. The incidence rates of hemodynamic instability were provided in nine studies, with hemodynamic instability occurring in 38.2% of the 1152 patients who underwent adrenalectomy for pheochromocytoma. Hemodynamic instability was defined broadly as any instability in blood pressure that could lead to inadequate blood flow to organs, with the precise definition differing among the 14 relevant studies. The median NOS score was 6 [6,7].

### 4.2. Meta-Analysis

We conducted a meta-analysis of baseline patient and tumor characteristics factors such as patient age, body mass index (BMI), tumor size, and urinary norepinephrine. Tumor size, age, and BMI were evaluated as continuous variables in the meta-analysis. Urinary norepinephrine was evaluated as a categorical variable.

### 4.3. Association of Tumor Size with Hemodynamic Instability

**Figure 2:**
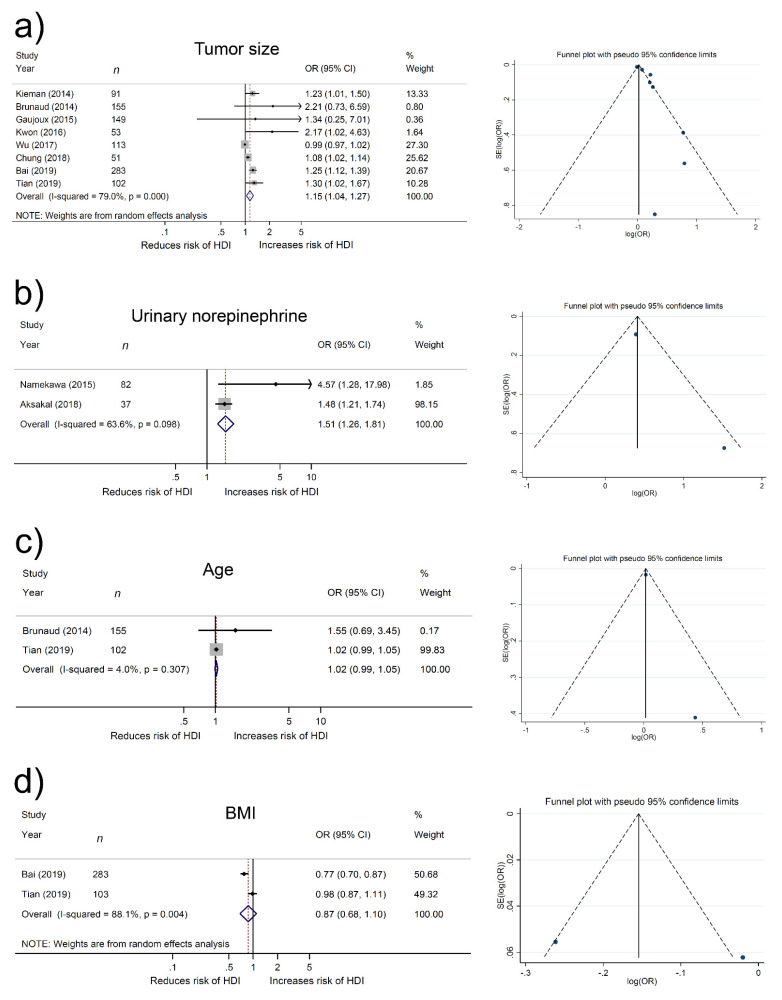
Forest and funnel plots showing the association of (**a**) tumor size, (**b**) urinary norepinephrine, (**c**) age, and (**d**) body mass index (BMI) with hemodynamic instability (HDI) in pheochromocytoma. OR, odds ratio.

Six studies (693 patients) provided data on the relationship between tumor size and hemodynamic instability. The forest plot (Figure 2a) showed that tumor size was significantly related to hemodynamic instability (pooled OR, 1.14; 95% CI 1.03–1.26; z = 2.57). The Cochrane Q test (Chi^2^ = 31.36; *p* < 0.001) and I^2^ test (I^2^ = 84.1%) showed significant heterogeneity. The funnel plot identified one study over the pseuo-95% CI (Figure 2a).

### 4.4. Association of Urinary Norepinephrine with Hemodynamic Instability

Two studies (119 patients) provided data on the relationship between urinary norepinephrine and hemodynamic instability. The Cochrane Q test (Chi^2^ = 2.75; *p* = 0.098) and I^2^ test (I^2^ = 63.6%) revealed no heterogeneity, so we used a fixed-effect model. The forest plot (Figure 2b) showed that urinary norepinephrine was significantly related to hemodynamic instability (pooled OR, 1.51: 95% CI 1.26–1.81; z = 4.50). The funnel plot identified no studies over the pseuo-95% CI (Figure 2b).

### 4.5. Association of Age with Hemodynamic Instability

Two studies (257 patients) provided data on the relationship between age and hemodynamic instability. The Cochrane Q test (Chi^2^ = 1.04; *p* = 0.307) and I^2^ test (I^2^ = 4.0%) revealed no heterogeneity, so we used a fixed-effect model. The forest plot (Figure 2c) showed that age was not significantly related to hemodynamic instability (pooled OR, 1.02: 95% CI 0.99–1.054; z = 1.16). The funnel plot identified no studies over the pseuo-95% CI (Figure 2c).

### 4.6. Association of BMI with Hemodynamic Instability

Two studies (386 patients) provided data on the relationship between BMI and hemodynamic instability. The forest plot (Figure 2d) showed that BMI was significantly related to hemodynamic instability (pooled OR, 0.87; 95% CI 0.68–1.10; z = 1.18). The Cochrane Q test (Chi^2^ = 8.38; *p* < 0.001) and I^2^ test (I^2^ = 88.1%) revealed significant heterogeneity. The funnel plot identified one study over the pseuo-95% CI (Figure 2d).

### 4.7. Other Factors Associated with Hemodynamic Instability

Surgical procedure (open surgery) [4], retroperitoneal approach [20], pre-operative beta blockade therapy [25], clinical symptoms [5], plasma epinephrine [7], plasma norepinephrine [7], plasma dopamine [7], plasma normetanephrine [25], urinary metanephrine and/or normetanephrine [5], familial disease [17], and the use of crystal/colloid fluid [23] were significantly associated with perioperative hemodynamic instability in one study each. Additionally, urinary epinephrine was evaluated in two studies. Both of those studies associated urinary epinephrine with hemodynamic instability [6,19], but the urinary epinephrine was evaluated as a categorical variable in one study [6] and as a continuous variable in the other [19], and these differences ruled out meta-analysis.

## 5. Discussion

To the best of our knowledge, our study is the first systematic review and meta-analysis to investigate the risk factors for perioperative hemodynamic instability in pheochromocytoma. Our meta-analysis utilized data from nine published articles with a combined patient population exceeding 900 patients. We used those data to evaluate factors associated with perioperative hemodynamic instability.

First, we found that tumor size (continuous variable) was associated with perioperative hemodynamic instability. Adrenal pheochromocytomas produce, store, release, and metabolize catecholamines, and larger pheochromocytomas involve considerable endocrine activity, which can result in severe hypertension during adrenalectomy. Notably, the resection of larger tumors has also been associated with chronically low circulating blood volume and an abrupt decrease in serum levels of catecholamines, which can cause severe hypotension after adrenalectomy. Moreover, in adrenalectomy, a larger tumor size entails difficulty and increases the degree of manipulation necessary to remove the tumor, which can also cause hemodynamic instability during operation [8]. To date, several reports have evaluated the relationship between tumor size and hemodynamic instability [4,6,7,17,19,21,22,23,24]. Our meta-analysis confirms that larger tumor size could be a risk factor for such instability.

We also found that urinary norepinephrine was associated with perioperative hemodynamic instability. Additionally, although we could not perform meta-analysis, previous studies have shown that urinary epinephrine can also be a risk factor for hemodynamic instability [6,19]. Adrenergic receptors are the final target for catecholamines, and those catecholamines are present in excess in patients with pheochromocytoma [27]. Thus, both urinary norepinephrine and urinary epinephrine are reasonable predictive factors for intra-operative hypertension and post-operative hypotension. However, both norepinephrine and epinephrine have overlapping but different effects on alpha- and beta-adrenergic receptors in various organs and systems [27]. Increased epinephrine causes a compensatory downregulation of beta-adrenergic receptors in the heart, which decreases cardiac contractility [28,29], while epinephrine and norepinephrine cause alpha adrenergic receptor mediated vasoconstriction, which can cause hypovolemia after tumor resection [27,30]. Thus, the classification of the dominant type of catecholamine might be important to perform the fluid replacement therapy for hypotension after tumor resection. Additionally, as Namekawa et al. reported that the urinary level of norepinephrine correlates with tumor size [6], further study will be required to confirm that these factors are independent risk factors of HDI.

We found several additional factors, including preoperative beta-blockade therapy and the surgical approach, were significantly associated with perioperative hemodynamic instability. However, those factors were excluded from meta-analysis because they were evaluated in only one study. In pheochromocytoma, an alpha blocker is generally used prior to pheochromocytoma resection, but the initiation of beta-blocker administration is sometimes considered for additional blood pressure control and control of tachyarrhythmias [31]. Thompson et al. reported that pre-operative beta-blockade therapy was the only independent predictor of postoperative hypotension [25]. Postoperative hypotension can result from a combination of the persistence of circulating antihypertensive drug and reversal of chronic vasospasm after tumor resection. Thus, preoperative medication should be carefully evaluated before the surgery.

Laparoscopic adrenalectomy has been shown to be a safe and feasible procedure for pheochromocytoma [32] and can be performed via the transperitoneal or retroperitoneal approach. Both of these approaches have been proven safe and effective [33,34]. Vorselaars et al. evaluated the effect of the surgical approach on hemodynamic instability during adrenalectomy and showed that retroperitoneal adrenalectomy carries greater risk of hypotension (MAP < 60 mmHg) than intraperitoneal adrenalectomy [20]. Although the mechanism of this difference has not yet been revealed and further examination will be required, the findings may be of considerable interest to surgeons and anesthesiologists.

This study represents the first systematic review and meta-analysis to assess risk factors for hemodynamic instability in patients with pheochromocytoma. The study has several limitations, however. First, selection (reporting) bias might lead to less frequent publication of negative findings. All studies in our meta-analysis were retrospective in design, increasing the risk of selection bias. Second, there was no consensus on cut-off values for the risk factors selected in our study. Most investigators selected their cut-off values based on their preferred statistical methods or on independently pre-defined biomarker cut-off values from the literature. Third, the studies did not use a uniform definition of perioperative hemodynamic instability. Indeed, vasoactive agents and volume therapy can directly influence the definition but usually not considered. In this context, recently, the hemodynamic instability score was proposed to quantify the overall degree of hemodynamic instability, and it may have future applications in both patient management and clinical research [35]. Fourth, the evaluated factors in each study enrolled in our meta-analysis were different between the studies. Finally, although the present study is limited to pheochromocytoma, our conclusion might also be valid for sympathetic paragangliomas. Future multi-center, large-scale epidemiological studies are needed to clarify risk factors for laparoscopic adrenalectomy in these patients.

## 6. Conclusions

Our meta-analysis indicates that tumor size and urinary norepinephrine are closely related to the occurrence of hemodynamic instability in patients with pheochromocytoma. These findings may be helpful to surgeons and anesthesiologists in cautiously preparing for perioperative hemodynamic instability in these patients.

## Figures and Tables

**Table 1:** The Studies’ Characteristics of All Included Articles.

First Author of Study and [Ref]	Country	Recruitment Period	*n*	D	Factors Evaluated (Cut off Values)	Significant Factors	Definition of HDI	NOS
Kiernan et al. [4]	USA	2002–2013	91	R	Procedure type (Open) Blockade type (Selective)	Procedure type, Blockade type	SBP > 200 mmHg	7
Kierman et al. [4]	USA	2002–2013	91	R	Tumor size (cm), Procedure type (Open)	Tumor size, Procedure type	Postoperative vasopressor requirement	7
Brunaud et al. [17]	USA	2002–2012	155	R	Age ≥ 45 (y) Tumor size ≥ 3 (cm) Familial disease	Familial disease	SBP ≥ 160 mmHg + MAP < 60 mmHg	7
Livingsone et al. [18]	France	2000–2017	134	R	Tumor size (mm) Diuretic before surgery (%) Beta-blocker before surgery (%)	(only univariate analysis) Tumor size Diuretic before surgery Beta-blocker before surgery	>10 hypo/hypertentive episodes where anesthesilogist had to use vasoactive	7
Gaujoux et al. [5]	France	1994–2011	149	R	Clinical symptom Urinary metanephrine and/or normetanephrine > 10 N Tumor size > 7 cm	preoperative HBP with clinical symptom Urinary metanephrine and/or normetanephrine >10 N	cumulative dose of norepinephrine > 5 mg	7
Namekawa et al. [6]	Japan	1999–2014	82	R	Tumor size > 6 cm Preoperative urinary Epinephrine > 200 µg/d Preoperative urinary norepinephrine > 600 µg/d	Tumor size Preoperative urinary epinephrine Preoperative urinary norepinephrine	Required continuous catecholamine support to maintain SBP ≥ 90 mmHg after adrenalectomy	7
Kwon et al. [19]	Korea	2000–2012	53	R	Tumor size (cm) SBP at presentation (mmHg) DBP at presentation (mmHg) Preoperative urinary epinephrine (µg/d) Preoperative urinary norepinephrine (µg/d) Preoperative urinary VMA (µg/d) Preoperative urinary metanephrine (µg/d)	Tumor size, Preoperative urinary epinephrine	SBP > 180 mmHg	6
Vorsellaars et al. [20]	Europe, USA, Canada	2000–2016	341	R	NR	retroperitoneal approach	MAP < 60 mmHg	6
Wu et al. [7]	China	2012–2016	113	R	Asymptomatic Diabetes mellitus ASA plasma norepinephrine (≥ULN) Plasma epinephrine Plasma dopamine Tumor size (cm)	Plasma epinephrine Plasma dopamine	MAP < 60 mmHg or required ≥ 30 consecutive minutes of catecholamine support intraoperatively	7
Chung et al. [21]	Korea	2005–2016	51	R	Clinical symptom Tumor laterality (right) Tumor size (cm) Perioperative urinary metanephrine (mg/day) Preoperative urinary VMA (mg/day)	Clinical symptom, Tumor size	SBP > 180 mmHg and MAP < 60 mmHg	6
Aksakal et al. [22]	Turkey	2008–2015	37	R	Age (y) Gender Side of Mass Operation duration (min) Duration of premedication (≥2 months) Urinary norepinephrine (≥2000 µg/24 h) Tumor size (≥6 cm)	Urinary norepinephrine, Tumor size	SBP > 200 mmHg or ≤ 90 mmHg	6
Bai et al. [23]	China	2007–2016	283	R	Intercept BMI (kg/m^2^) Coronary heart disease Use of crystal/colloid fluid Tumor size (cm)	Intercept BMI Coronary heart disease Use of crystal/colloid fluid Tumor size	SBP > 200 mmHg + MAP < 60 mmHg or Required catecholamine to maintain SBP	6
Tian et al. [24]	China	2001–2018	102	R	DFD < 14 d Age (y) Tumor size (cm) BMI (kg/m^2^) Surgical approach Tumor location Biochemical positive	Tumor size	SBP > 200 mmHg, SBP > 130% of basic SBP, SBP < 80 mmHg, SBP < 70% of basic SBP HR > 120 bpm	6
Thompson et al. [25]	UK	2007–2014	52	R	Tumor size (cm) Plasma norepinephrine (>3500 pmol/L) Laparoscopic approach Epidural analgesia	Plasma normetanephrine level	SBP > 200 mmHg	6
Thompson et al. [25]	UK	2007–2014	45	R	Tumor size (cm) Preoperative beta-blockade Postoperative fluid volume (<24 h, L)	Preoperative beta-blockade	SBP < 90 mmHg	6
Buisset et al. [26]	Canada	1992–2013	88	R	Preoperative penoxybenzamine dose (mg) Preoperative SBP (mmHg) Intraoperative vasopression use magnesium use	(only univariate analysis) Preoperative penoxybenzamine dose Preoperative SBP Intraoperative vasopression use magnesium use	Required pressor amines postoperatively	6

ASA, American Society of Anesthesiologists Physical Status Classification System; BMI, body mass index; D, design; DBP, diastolic blood pressure; DFD, duration of final dose; HBP, high blood pressure; HR, heart rate; HDI, hemodynamic instability; MAP, mean arteral pressure; *n*, number; NOS, Newcastle-Ottawa Scale; NR, not reported; R, retrospective; SBP, systolic blood pressure; ULN, upper limit of normal; VMA, vanillylmandelic acid.

**Table 2:** Patients’ Characteristics.

	Pt No.			Sex (M; F)			Age (y)			BMI (kg/m^2^)		
First Author of Study and [Ref]	Total	HI	Non-HI	Total	HI	Non-HI	Total	HI	Non-HI	Total	HI	Non-HI
Kiernan et al. [4]	91	NR	NR	43; 48	NR	NR	52	NR	NR	27.5	NR	NR
Brunaud et al. [17]	155	NR	NR	68; 87	NR	NR	52	NR	NR	25.8	NR	NR
Livingsone et al. [18]	88	NR	NR	42; 46	NR	NR	50	NR	NR	NR	NR	NR
Gaujoux et al. [5]	149	13	136	48; 101	7; 6	41; 95	NR	58	53	NR	24.2	23.2
Namekawa et al. [6]	73	34	39	30; 43	15; 19	15; 24	48	46	53	21	21	22
Kwon et al. [19]	53	33	20	28; 25	15; 18	13; 7	47.5	47.5	47.5	23.3	23.1	23.5
Vorsellaars et al. [20]	341	169	172	149; 192	NR	NR	49	NR	NR	24.9	NR	NR
Wu et al. [7]	123	54	69	48; 75	21; 33	27; 42	46	47	45	24	23	24
Chung et al. [21]	51	25	26	25; 26	NR	NR	52	NR	NR	23.3	NR	NR
Aksakal et al. [22]	37	13	24	14; 23	5; 8	9; 15	39.3	40.6	38.6	NR	NR	NR
Bai et al. [23]	283	74	209	141; 142	31; 43	110; 99	52.4	54	51.9	23.5	21.9	24.1
Tian et al. [24]	102	NR	NR	47; 55	NR	NR	43.1	NR	NR	47	NR	NR
Thompson et al. [25]	42	25	17	11; 31	6; 19	5; 12	51	52	49	26	26	27
Thompson et al. [25]	45	21	24	12; 33	7; 14	5; 19	51	50	51	27	25	29
Buisset et al. [26]	134	NR	NR	62; 72	NR	NR	51	NR	NR	24	NR	NR

HI: hemodynamic instability; NR, not reported; Pt, patient.

**Table 3:** The characteristics of tumor and surgical approach.

	Tumor Size (cm)			Tumor Laterality (Right)			Surgical Approach (Laparoscopic Surgery)			Clinical Symptom		
First Author of Study and [Ref]	Total	HI	Non-HI	Total	HI	Non-HI	Total	HI	Non-HI	Total	HI	Non-HI
Kiernan et al. [4]	4	NR	NR	NR	NR	NR	71 (78%)	NR	NR	NR	NR	NR
Brunaud et al. [17]	4.5	NR	NR	84 (54.2%)	NR	NR	100%	100%	100%	Palpitation: 60 (39%) Sweating: 60 (39%) Headaches: 55 (35%)	NR	NR
Livingsone et al. [18]	4.2	NR	NR	NR	NR	NR	43 (48.9%)	NR	NR	NR	NR	NR
Gaujoux et al. [5]	NR	6.5	5	55 (36.9%)	7 (53.8%)	58 (42.6%)	149 (100%)	13 (100%)	136 (100%)	45 (30.2%)	9 (69.2%)	36 (26.5%)
Namekawa et al. [6]	4.6	5	4.2	NR	NR	NR	100%	100%	100%	35 (47.9%)	20 (58.8%)	15 (38.5%)
Kwon et al. [19]	5.59	6.48	4.11	24 (45.3%)	13 (36.4%)	11 (55.0%)	44 (83.0%)	26 (78.8%)	18 (90%)	25 (47.2%)	18 (54.5%)	7 (35.0%)
Vorsellaars et al. [20]	4.17	NR	NR	175 (51%)	NR	NR	NR	NR	NR	NR	NR	NR
Wu et al. [7]	4.6	4.9	4.4	NR	NR	NR	123 (100%)	100%	100%	21 (17.1%)	5 (9%)	16 (23%)
Chung et al. [21]	5.6	NR	NR	22 (43.1%)	NR	NR	51 (100%)	100%	100%	26 (51%)	NR	NR
Aksakal et al. [22]	<6 cm: 24 (64.7%)	<6 cm: 11 (84.6%)	<6 cm: 13 (54.1%)	21 (56.8%)	8 (61.5%)	13 (54.2%)	NR	NR	NR	NR	NR	NR
Bai et al. [23]	5.5	6.5	5.2	141 (49.8%)	35 (47.3%)	106 (50.7%)	132 (46.6%)	32 (43.2%)	100 (47.8%)	NR	NR	NR
Tian et al. [24]	5	NR	NR	57 (55.9%)	NR	NR	63 (61.8%)	NR	NR	NR	NR	NR
Thompson et al. [25]	4.4	4.8	3.8	NR	NR	NR	15 (35.7%)	13 (52%)	2 (13%)	NR	NR	NR
Thompson et al. [25]	4.4	5	3.8	NR	NR	NR	18 (40%)	6 (30%)	12 (50%)	NR	NR	NR
Buisset et al. [26]	3.96	NR	NR	71 (53.0%)	NR	NR	134 (100%)	NR	NR	NR	NR	NR

HI: hemodynamic instability; NR, not reported.

## Data Availability

All data accessed are available in the article.
